# Everolimus in Heart Transplantation: An Update

**DOI:** 10.1155/2013/683964

**Published:** 2013-12-05

**Authors:** Stephan W. Hirt, Christoph Bara, Markus J. Barten, Tobias Deuse, Andreas O. Doesch, Ingo Kaczmarek, Uwe Schulz, Jörg Stypmann, Assad Haneya, Hans B. Lehmkuhl

**Affiliations:** ^1^Department of Cardiothoracic Surgery, University Hospital Regensburg, Franz-Josef-Strauß-Allee 11, 93053 Regensburg, Germany; ^2^Division of Cardiovascular,Thoracic and Transplantation Surgery, Hannover Medical School, Carl-Neuberg-Straße 1, 30625 Hannover, Germany; ^3^Department of Cardiac Surgery, University Hospital Leipzig, Heart Center, 04289 Leipzig, Germany; ^4^Department of Cardiovascular Surgery, University Heart Center Hamburg, Martinistr 52, 20246 Hamburg, Germany; ^5^Department of Cardiology, University Hospital Heidelberg, Im Neuenheimer Feld 410, 69120 Heidelberg, Germany; ^6^Department of Cardiac Surgery, Munich Transplantation Center, Klinikum Großhadern LMU, Marchioninistraße 15, 81377 Munich, Germany; ^7^Department of Thoracic and Cardiovascular Surgery, Heart and Diabetes Center North Rhine-Westphalia, Georgstraße 11, 32545 Bad Oeynhausen, Germany; ^8^Department of Cardiovascular Medicine, Division of Cardiology, Münster University Hospital, Albert-Schweitzer-Straße 33, 48149 Münster, Germany; ^9^Department of Cardiovascular Surgery, University Hospital Kiel, Arnold-Heller-Straße 7, 24105 Kiel, Germany; ^10^Department of Cardiovascular and Thoracic Surgery, German Heart Institute Berlin, Augustenburger Platz 1, 13353 Berlin, Germany

## Abstract

The evidence base relating to the use of everolimus in heart transplantation has expanded considerably in recent years, providing clinically relevant information regarding its use in clinical practice. Unless there are special considerations to take into account, all *de novo* heart transplant patients can be regarded as potential candidates for immunosuppression with everolimus and reduced-exposure calcineurin inhibitor therapy. Caution about the use of everolimus immediately after transplantation should be exercised in certain patients with the risk of severe proteinuria, with poor wound healing, or with uncontrolled severe hyperlipidemia. Initiation of everolimus in the early phase aftertransplant is not advisable in patients with severe pretransplant end-organ dysfunction or in patients on a left ventricular assist device beforetransplant who are at high risk of infection or of wound healing complications. The most frequent reason for introducing everolimus in maintenance heart transplant patients is to support minimization or withdrawal of calcineurin inhibitor therapy, for example, due to impaired renal function or malignancy. Due to its potential to inhibit the progression of cardiac allograft vasculopathy and to reduce cytomegalovirus infection, everolimus should be initiated as soon as possible after heart transplantation. Immediate and adequate reduction of CNI exposure is mandatory from the start of everolimus therapy.

## 1. Introduction

The mammalian target-of-rapamycin inhibitor (mTOR) everolimus has been licensed in Europe since 2004 for the prevention of organ rejection in adult patients at low to moderate immunological risk receiving an allogeneic kidney, liver or heart transplant. Everolimus is currently the only mTOR inhibitor approved for use in heart transplantation. It was developed to improve the pharmacokinetics of the mTOR inhibitor sirolimus through a stable 2-hydroxyethyl chain substitution at position 40 of the sirolimus molecule [1]. This change confers a shorter half-life, permitting faster reduction or elimination of everolimus exposure and obviating the need for a loading dose.

In a pivotal double-blind phase 3 (B253) trial in *de novo* heart transplant recipients published in 2003, Eisen et al. demonstrated that everolimus provided equivalent immunosuppressive efficacy to azathioprine [2]. Inhibition of vascular smooth muscle cell proliferation by everolimus reduced intimal thickening and lowered the incidence of cardiac allograft vasculopathy (CAV). Based on these findings and early clinical experience in Germany and Austria, recommendations on the use of everolimus in heart transplantation were developed at two expert meetings held in 2004 [3] and 2006 [4]. Since then, the evidence base relating to everolimus in heart transplantation has expanded substantially with additional randomized studies in *de novo* [5] and maintenance [6–8] heart transplant patients. Recently, results from the 24-month, international, randomized, open-label study A2310 [9] have been published, refocusing attention on the use of everolimus in *de novo* heart transplant patients. This paper considers the current data set and considers the implications for use of everolimus in this setting.

## 2. Everolimus in Heart Transplant Recipients: The Key Studies

### 2.1. Study Designs

The key studies evaluating the *de novo* use of everolimus following heart transplantation and their primary endpoints, are summarized in Table 1. In a 24-month, multicenter, randomized, double-blind, double-dummy, phase 3 study (B253), the efficacy, safety, and tolerability of two fixed doses of everolimus (1.5 and 3.0 mg/day) were compared to azathioprine in 634 *de novo* heart transplant recipients [2]. All patients received a triple drug regimen that included standard-dose cyclosporine (CsA) and corticosteroids. In the multicenter, randomized A2411 trial (*n* = 176), the immunosuppressive regimen was changed to concentration-controlled everolimus (initial dose 1.5 mg/day, target trough level *C*_0_ 3–8 ng/mL) and reduced-exposure CsA to examine whether renal toxicity was reduced compared to mycophenolate mofetil (MMF, 3 g/day) with standard CsA [5]. Both groups received steroids and antibody induction therapy according to local practice. In an observational study by Lehmkuhl et al., everolimus (*C*_0_ 3–8 ng/mL) with low-exposure CsA was compared to MMF (mean dose 1.25–2.5 g/day) in combination with standard CsA in 52 *de novo* heart transplant patients [10]. All patients received induction with antithymocyte globulin (two doses of 2.5 mg/kg) and steroids. Mean CsA *C*_0_ decreased by 58% from week 2 to month 12 in the everolimus group versus 35% in the MMF cohort (mean [SD] at month 12: 101 [26] ng/mL) versus 160 [41] ng/mL).

The A2310 trial was an international, open-label, 24-month study in which 721 *de novo *heart transplant recipients were randomized to (i) standard everolimus trough concentration (3–8 ng/mL), to (ii) high everolimus trough concentration (6–12 ng/mL) both with reduced-dose CsA, or to (iii) MMF 3 g/day with standard-dose CsA [9]. All patients received corticosteroids with or without induction according to center practice. Randomization to the high everolimus trough concentration group (6–12 ng/mL) was stopped prematurely due to higher early mortality, and data from this group were not analyzed in detail. The primary efficacy endpoint was the composite efficacy failure (biopsy-proven acute rejection of ISHLT grade ≥ 3A, acute rejection episodes associated with hemodynamic compromise, graft loss/retransplant, death, and loss to followup) and the main secondary efficacy endpoint was the incidence rate of graft loss/retransplant, death, or loss to followup, both at month 12 [9].

### 2.2. Efficacy Results

According to the primary efficacy endpoints, everolimus was significantly more efficacious than azathioprine at month 6 aftertransplant when both agents were administered with standard-dose CsA (B253) [2], and noninferior when given with reduced-exposure CsA compared to MMF plus standard-exposure CsA at months 12 and 24 aftertransplant (A2310) [9] (Table 2). The German single-center observational study by Lehmkuhl et al. indicated that a low initial CsA target trough range of 200–250 ng/mL during the first month aftertransplant, subsequently downtitrated to achieve a reduction of 58% by month 12, is feasible without loss of immunosuppressive efficacy [10].

In B253, the incidences of graft loss and death were comparable between treatment groups [2]. In the A2310 trial, the combination of highly-exposure everolimus (target *C*_0_ 6–12 ng/mL) with CsA and MMF was associated with increased mortality, leading to discontinuation of recruitment to that study arm. In the standard-exposure everolimus group, mortality was similar to the control arm only in the absence of induction therapy. Increased infection-related mortality was observed during the first three months aftertransplant in patients receiving standard-exposure everolimus in conjunction with antithymocyte globulin (Thymoglobulin) induction. Further subanalyses revealed an association of deaths in the everolimus group with the use of a left ventricular assist device (LVAD) beforetransplant, and that virtually all deaths in patients with LVAD and Thymoglobulin induction occurred in German centers. The German procedure for selecting highly urgent heart transplant recipients for preferred allocation results in a very high-risk population; for example, LVAD patients are only allocated to a donor very urgently in the event of technical failure, relapsing strokes, or LVAD infection. If these LVAD patients, often with specific risks such as concomitant infection, receive Thymoglobulin induction plus early introduction of a mTOR inhibitor, the intensity of immunosuppression can become supratherapeutic. In these patients, everolimus should not be initiated until wound healing is complete and any bacterial or fungal infections have been cleared. By month 24, the mortality rate in the A2310 study was similar in the everolimus and MMF groups (10.6% versus 9.2%, resp.). Other efficacy endpoints were also similar between the two treatment groups, consistent with earlier data from the A2411 trial in *de novo* heart transplant patients (Table 2).

### 2.3. Safety Profile

In most cases, the side effects of everolimus (e.g., dyslipidemia, elevated creatine kinase, acne, aphthous stomatitis, edema, pneumonia, proteinuria, leukopenia, and thrombocytopenia) are manageable with the adjustment of concomitant medication or reduction of everolimus dose or with interruption of everolimus therapy for a few days [11].

Due to their antiproliferative properties, mTOR inhibitors can impair wound healing after surgery [12]. Clinical evidence regarding an effect on wound healing in heart transplantation is mixed [5, 9, 13]. Randomized studies indicate an elevated incidence of pericardial and possibly pleural effusion (see Supplementary Table 1 in Supplementary Material available online at http://dx.doi.org/10.1155/2013/683964). In the A2310 study, pericardial effusions were most frequent in the everolimus treatment group (43.4% versus 28.4% with MMF at month 12, *P* < 0.001), but rates of pericardial tamponade, pleural effusions, sternal and nonsternal wound healing complications, and wound infections were similar between groups [5, 9, 13] (Supplementary Table 1). The difference in pericardial effusions contributed to a higher overall rate of study drug discontinuation due to adverse events with everolimus versus MMF at 12 months (29.7% versus 19.0%) although this diminished by month 24 (33.3% versus 25.7%) [10]. The ongoing EVERHEART study (NCT01017029), which is being undertaken in a *de novo* heart transplant population randomized to receive everolimus immediately or with a delay of 4–6 weeks, includes pericardial effusion as a prespecified endpoint [14].

Viral infections were less frequent with everolimus versus MMF in the A2310 trial, largely accounted for by a lower rate of cytomegalovirus (CMV) infection in everolimus-treated patients (8.2% versus 20.5% with MMF at 12 months, *P* < 0.001; 9.3% versus 23.9% at month 24, *P* < 0.001). These results substantiate similar findings in the A2411 study [5], in B253 study [2], and a recent pooled analysis [15]. The consistent reduction in CMV infection with everolimus versus azathioprine or MMF [2, 5, 9] is independent of CMV prophylaxis and donor/recipient serostatus [9]. Other viral infections such as herpes simplex virus, Epstein-Barr virus, polyoma virus, and herpes zoster virus may be lowered by everolimus, but studies have not been designed with these infections as predefined endpoints. Of note, although viral infections are reduced, bacterial or fungal infections may be more frequent with everolimus, and avoiding overimmunosuppression is critical to reduce this risk.

### 2.4. Renal Function

Neither the A2310 study [9] nor the A2311 trial [5] showed a renal benefit for everolimus versus MMF (Supplementary Table 2). Indeed, noninferiority of renal function for everolimus versus MMF was not shown in the A2310 study since the lower limit of the confidence interval was lower than the prespecified margin of −10 mL/min/1.73 m^2^ (the difference in mean eGFR was −5.55 mL/min/1.73 m^2^, 97.5% CI [−10.9, −0.2]) [9]. This was probably due to the absence of CsA dose reduction during the first month aftertransplant and subsequent nonadherence to targets for CsA reduction in the everolimus group. It is interesting to note that from month 1 to month 12, when CsA target levels were lower in everolimus-treated patients, the decline in eGFR was smaller with everolimus versus MMF (−8.6 versus −14.6 mL/min/1.73 m^2^, *P* = 0.009) [9]. 

Converting maintenance heart transplant patients from a standard CNI regimen to everolimus with reduced CNI therapy can offer a significant improvement in renal function, as demonstrated in the randomized NOCTET study [6] and during single-center experience [16], even when administered at a low dose [17], although conflicting data exist [8]. CsA dose must be reduced stepwise compared to standard dosing in the presence of everolimus, which can be undertaken without loss of efficacy [18], or the CsA reduction is inadequate to protect renal function. In the event of CNI-related nephrotoxicity, early switch to a mTOR inhibitor appears advisable since the positive effects on renal function are more pronounced if conversion is performed in the first year, although no specific time limit has been established. In the SHIRAKISS trial of 34 maintenance patients with renal dysfunction who were between one and four years aftertransplant, conversion to everolimus with a 70% reduction in CsA exposure only improved renal function in patients without proteinuria at the time of conversion [8]. Patients with proteinuria continued to show renal deterioration despite the switch to everolimus therapy. The decision on timing needs to take into account the fact that CNI therapy may be necessary for the first nine months after heart transplantation. Most side effects in maintenance patients occur within six months after starting everolimus and may necessitate a temporary switch back to CNI therapy. In patients with steroid-resistant recurrent myocardial rejection, permanent reintroduction of low-dose CNI may be required.

There is widespread experience in German centers of CNI withdrawal and long-term CNI-free immunosuppression using everolimus in maintenance patients after heart transplantation. Stypmann et al. described a cohort of 60 patients switched to a CNI-free regimen in response to deteriorating renal function, recurrent rejection, or side effects under CNI-based therapy [19]. After 24 months, renal function had improved significantly (mean (SD) creatinine clearance (Cockcroft-Gault) 41.8 [22]  mL/min versus 48.6 [21.8] mL/min at baseline, *P* < 0.001).

### 2.5. Cardiac Allograft Vasculopathy

The B253 study of everolimus versus azathioprine in *de novo* heart transplant patients first indicated that everolimus may inhibit the development of CAV [2]. Intravascular ultrasonography (IVUS) studies showed a significant reduction of the average increase of the maximal intimal thickness (MIT) from baseline to month 12 aftertransplant in patients receiving everolimus compared to azathioprine and a significantly lower incidence of CAV (defined as an increase in MIT ≥ 0.5 mm) (Table 3). These findings are highly relevant since MIT at 12 and 24 months after heart transplantation predicts subsequent major adverse cardiac events and death [21, 22]. In the A2310 study, IVUS data at month 12 confirmed that the mean increase in MIT was smaller with everolimus than MMF, accompanied by a lower incidence of protocol-defined CAV (Table 3). All other predefined IVUS endpoints were also significantly in favor of everolimus [9]. This benefit was observed despite higher mean levels of total cholesterol in everolimus-treated patients [9].

According to ISHLT guidelines everolimus, sirolimus as tolerated, or MMF should be a part of the immunosuppression regimen after heart transplantation to reduce the onset and progression of CAV [23]. mTOR inhibition can be substituted for MMF or azathioprine in patients who develop CAV, although data are lacking regarding the effect of late conversion to mTOR inhibition on CAV progression.

### 2.6. Posttransplant Malignancy

mTOR inhibitors exert a direct antineoplastic effect by the inhibition of the phosphatidylinositol-3-kinase (PI3K) pathway and by sensitization of tumor cells to apoptosis via inhibition of the p53-induced p21 expression regulating abnormal cellular proliferation and differentiation [24]. A randomized, double-blind, phase 3 trial has demonstrated significantly better progression-free survival in patients with metastatic renal cell carcinoma who received everolimus compared to placebo (RECORD-1) [25, 26]. Everolimus is licensed for the treatment of advanced renal cell carcinoma and for advanced breast cancer and is currently under investigation for the management of other types of malignancy.

There are case reports describing regression of Kaposi's sarcoma [27] and malignant neoplasia [28, 29] in kidney transplant recipients following conversion from CNI to everolimus, and Tiberio et al. have described significant regression of cardiac rhabdomyoma in a patient receiving everolimus [30]. Kusuki et al. reported a case of successful management of diffuse large B-cell lymphoma 29 months after cardiac transplantation in a 47-month-old boy using minimized CsA in combination with everolimus following rituximab treatment and chemotherapy [31]. Conversion from CNI to everolimus therapy to control malignancy following heart transplantation would seem a reasonable therapeutic approach, particularly for Kaposi's sarcoma, nonmelanoma skin cancer, and renal cell carcinoma [32] but robust data are lacking. The ongoing multicenter, randomized CERTICOEUR trial (NCT00799188) is comparing the development of new skin cancers in 159 heart transplant patients suffering recurrent skin cancer receiving everolimus and reduced or discontinued CNI therapy versus standard CNI therapy. Importantly, evidence is also growing for a protective role of mTOR inhibitors on the risk of developing new malignancies or nonskin solid tumors following kidney transplantation [32]. Data are awaited in heart transplant recipients.

### 2.7. Pediatric Heart Transplant Recipients

Minimization of steroids and exposure to CNIs are especially vital in children to reduce the risk of metabolic disorders, renal dysfunction, and cancer. While early withdrawal of steroids is a well established strategy in pediatric transplant recipients, there is only limited experience with-reduced-CNI or CNI-free regimens [33, 34]. Everolimus is not currently licensed in children and its use in pediatric heart transplant patients is largely restricted to high-volume centers [35]. There are no randomized trials. Behnke-Hall et al. have published their experience of switching from CNI therapy to everolimus in 28 children with poor renal function (eGFR < 75 mL/min/1.73 m^2^) at a median of 9.81 years following heart transplantation [36]. All patients were also receiving azathioprine or MMF (those on azathioprine were converted to MMF before the switch to everolimus). In this series, median eGFR increased significantly from the time of conversion (47.81 mL/min/1.73 m^2^) to six months (63.1 mL/min/1.73 m^2^) and 12 months (64.8 mL/min/1.73 m^2^) after conversion (both *P* < 0.05), although three patients experienced rejection and side effects were common. More extensive experience is available in pediatric kidney transplantation [37–39], indicating that *de novo* use of everolimus with CsA offers effective immunosuppression and good renal function to three years aftertransplant [39]. Analyzing protocol biopsies at six months after renal transplantation, Kanzelmeyer et al. found a significantly lower number of pathological changes in patients treated with everolimus and low-dose CNI compared to standard CNI-based treatment [40].

Due to greater oral clearance, pediatric patients may require higher dosages than adults when adjusted according to weight and body surface area or shorter dosing intervals. In their series of pediatric maintenance heart transplant recipients converted from CNI therapy to everolimus, Behnke-Hall and colleagues targeted an everolimus trough concentration of 5–8 ng/mL, with a mean (SD) starting dose of 0.07 (0.05) mg/kg given in two divided doses. CNI was withdrawn once the everolimus trough concentration was within range. The mean (SD) change in everolimus dose after three months was 0.15 (0.17) mg/kg. In this cohort of 28 patients, three patients experienced acute rejection following switch and three developed infections. Experience from pediatric kidney transplantation suggests that an everolimus trough concentration of 4–6 ng/mL for the first 6 months aftertransplant and then 3–5 ng/mL thereafter [41] or more simply a trough level of ≥3 ng/mL [42], with reduced-concentration CsA, may be adequate. Off-label use in pediatric liver transplant patients is limited, but initial data suggest that a trough concentration in the range 4–6 ng/mL with reduced-exposure CsA may be sufficient when introducing everolimus as rescue therapy for chronic graft failure [43].

## 3. Everolimus Administration and Dosage Regimens

Everolimus acts synergistically with CsA, such that CsA exposure can be reduced without the loss of efficacy. To avoid the risk of potentiating CNI-related nephrotoxicity, CsA exposure should be reduced in the presence of everolimus. Lehmkuhl et al. reported a reduction in mean CsA trough concentration of 47% at two weeks and 58% at 12 months after transplantation [10] compared to standard dose. A drug-drug interaction between everolimus and tacrolimus, by which everolimus decreases tacrolimus oral bioavailability in a dose-dependent manner [44], means that tacrolimus dose reductions should be smaller than those required for CsA, particularly during the early posttransplant phase, to avoid rejection.

In CNI-free regimens, everolimus should be used in combination with mycophenolic acid.

Everolimus has a shorter half-life than sirolimus (28 hours versus 62 hours) with a more rapid time to steady state (4 days versus 5–7 days) and as a result does not require a loading dose [45–50] (Supplementary Table 3). It is administered twice daily together with the concomitant immunosuppressive medication. Stable trough blood levels (3–8 ng/mL) can be obtained after approximately 3–7 days and should be monitored 1–2 times a week initially, then weekly for the following two months, and every 2–4 weeks thereafter. If the everolimus dose or concomitant medication is changed, the frequency of monitoring should be increased until steady state is achieved. The daily dose should not exceed 3.0 mg even if the target trough concentration is not achieved other than in a very few instances (e.g., in patients receiving comedication that induces enzymatic induction), when a higher dose may be appropriate for a limited period.

## 4. Selection of Patients for Everolimus Therapy

### 4.1. *De Novo* Heart Transplant Recipients

Unless there are special considerations to take into account, all *de novo* heart transplant patients can be considered potential candidates for everolimus-based immunosuppression. Caution should be exercised in certain categories of patients, however, such as those at risk of severe proteinuria, poor wound healing, or patients who have uncontrolled severe hyperlipidemia or a highly elevated risk of infection (Table 4). The potential risk of impaired wound healing and fluid retention at operative sites indicates that delayed initiation of everolimus after transplantation (e.g., approximately 8–14 days) or after other surgical interventions may be helpful although such an approach has shown no benefit in kidney transplantation [51]. Delayed introduction of everolimus has not yet been systematically explored in heart transplantation but may be most relevant in heart transplant patients with risk factors for poor healing, particularly obesity or diabetes, and in patients with previous coronary artery bypass grafting with bilateral harvesting of the mammarial arteries or those undergoing re-thoracotomy early after transplantation [13, 52].

Routine use of everolimus in the early phase post-transplant is not appropriate in patients with severe end-organ dysfunction prior to transplantation or in patients on LVAD who are considered to be at high risk for infection or wound healing complications. This is especially the case if LVAD infection was the indication for heart transplantation and Thymoglobulin is used as induction therapy (or, potentially, to treat early rejection) since the findings of A2310 suggest that this combination may lead to overimmunosuppression.

### 4.2. Maintenance Heart Transplant Recipients

The most frequent reason for introducing everolimus to a maintenance immunosuppression regimen is to support the minimization or withdrawal of CNI therapy, for example, in response to impaired renal function or malignancy. In such cases, conversion to a combination of everolimus with mycophenolic acid plus steroids is usually an appropriate option. Additionally, an antimetabolite agent may be switched to everolimus at any time after transplantation with the most likely indications being repeated rejection or adverse events caused by azathioprine or mycophenolic acid. Theoretically, conversion from an antimetabolite to everolimus could be used to inhibit the development of CAV progression, but currently there is only very limited clinical experience to suggest that late switch is beneficial in patients with established CAV.

When everolimus is introduced to replace an antimetabolite agent (mycophenolate or azathioprine), the dose of CsA should be reduced immediately at the time of everolimus initiation. For a short period (approximately four days), everolimus should be given in addition to the antimetabolite, which is then withdrawn as soon as an adequate everolimus trough concentration (*C*_0_ 3–8 ng/mL) is achieved. Close surveillance by echocardiography and at outpatient visits is important during the first weeks after conversion to ensure that acute rejection, while unlikely, is detected promptly.

In cases where everolimus is introduced to substitute for CNI therapy in patients receiving mycophenolic acid, everolimus should be started at a dose of 0.75 mg b.i.d., with stepwise reductions in CNI dose. Once the everolimus trough concentration is in the range 3–8 ng/mL, the CNI is withdrawn. In patients receiving everolimus with CNI and a CNI-free regimen is sought, MMF can be introduced at a dose of 1.0–1.5 g b.i.d. with stepwise withdrawal of CNI starting approximately one week later. In the early period after CNI withdrawal, close observation of allograft function by echocardiography and endomyocardial biopsy coupled with monitoring of everolimus and mycophenolic acid trough concentrations is very important. Patients who are receiving azathioprine and CNI also need a stepwise approach to CNI discontinuation. First, azathioprine is replaced by everolimus with a simultaneous reduction in CNI dose. Azathioprine is discontinued as soon as adequate everolimus trough concentrations are reached. In a second step, several weeks later, the CNI dose is reduced stepwise while mycophenolic acid is introduced. Close monitoring of allograft function is again mandatory.

## 5. Drug Interactions

Everolimus interacts with cytochrome P450 (CYP) enzymes 3A4, 3A5, and 2C8 [53]. Drugs which influence the CYP3A pathway, in particular, affect everolimus metabolism. Concomitant administration of some CYP3A4 inhibitors (e.g., azithromycin, erythromycin, ketoconazole, and itraconazole) induces 18–74% reduction in everolimus clearance, resulting in an increased maximum concentration and prolonged everolimus half-life, while others (e.g., calcium channel blockers, quinolones, and trimethoprim-sulfamethoxazole) have no relevant effect. CYP3A inducers (rifampicin, phenytoin, and carbamazepine) decrease everolimus blood concentration to varying degrees. CsA is metabolized via the CYP3A isoenzyme system and has been shown in single-dose healthy volunteer studies to increase everolimus blood concentration [54] but the steady-state pharmacokinetics of CsA are not influenced by coadministration of everolimus. A reduction in CsA exposure is necessary to avoid CNI-related nephrotoxicity in combination with everolimus.

The combination of tacrolimus and everolimus for prophylaxis of acute rejection after heart transplantation is administered in selected patients in some German centers, although this remains off-label use. There is evidence from kidney transplantation that co-administration of everolimus with tacrolimus reduces tacrolimus exposure [44]. Therefore, tacrolimus dose reduction is considered necessary, although to a lesser extent than for CsA. Tacrolimus does not influence everolimus blood levels, such that higher doses of everolimus are required than those in CsA-treated patients to maintain therapeutic blood levels of everolimus [55]. Tacrolimus is as effective as CsA in combination with everolimus after heart transplantation, and the incidence of serious hypertriglyceridemia is similar [56].

## 6. Management of Adverse events

Everolimus trough blood concentrations in the range 3–8 ng/mL are well tolerated and associated with a low incidence of side effects, but higher levels are not well tolerated. If everolimus trough concentration exceeds 10 ng/mL, an immediate dosage reduction is likely to be necessary since in addition to a high incidence of everolimus-specific side effects there is an increased risk of over-immunosuppression. Most adverse events are not lifethreatening and are responsive to treatment. In clinical practice, preventive measures, optimal screening, and management of side effects should be routine. Experienced-based algorithms may help to avoid the need for everolimus discontinuation.

Management strategies for specific types of everolimus-related adverse events in heart transplant recipients have been discussed in detail elsewhere [4, 11] and key aspects are summarized in Table 5. Routine comedication with lipid-lowering medication is essential in heart transplant patients receiving a mTOR inhibitor. Statin therapy is standard, but in view of the known potential for drug-drug interactions between drugs that affect CYP3A metabolism of everolimus, agents that do not interact with CYP450 should be selected, such as pravastatin, fluvastatin, or fibrates.

## 7. Discontinuation of Everolimus

Discontinuation of everolimus in heart transplant recipients is associated with a decline in renal function [57] but withdrawal or temporary interruption may be necessary if severe everolimus-related side effects cannot be managed or if surgery is planned. Everolimus can be replaced by MMF using a stepwise switch. In the event of surgery, this stepwise process should be timed to ensure that everolimus is withdrawn at least seven days before the operation is scheduled. Since adequate blood concentration of MMF requires several days to achieve, overlap of everolimus and MMF administration is advisable for approximately four days. The CNI blood level is likely to increase during everolimus withdrawal and both blood concentrations and renal function should be monitored closely during and after discontinuation. After the side effects have resolved or wound healing is complete, reintroduction of everolimus can be considered.

In patients receiving CNI-free immunosuppression, the risk of postoperative infection must be carefully balanced with the risk of renal function impairment associated with the reintroduction of CNI. For elective major thoracic, abdominal, and retroperitoneal surgery, stepwise reintroduction of CNI in combination with MMF and withdrawal of everolimus is appropriate. This switch should be undertaken approximately two weeks before surgery with reconversion to a CNI-free regimen as soon as wound healing is completed.

## 8. Conclusions

The efficacy of everolimus at a trough concentration of 3–8 ng/mL in combination with reduced-exposure CsA is noninferior to MMF plus full-exposure CsA up to two years after heart transplantation. Data regarding concomitant use of everolimus with tacrolimus remain limited. The side effects which are potentially associated with the use of mTOR inhibitors do not represent a major threat in the clinical situation. In addition, when administered in combination with MMF, everolimus offers the option of CNI-free immunosuppression in selected patients beyond the first year after heart transplantation.

Recent concerns about early increased mortality in the everolimus groups of the A2310 study can be explained by overimmunosuppression in patients with LVAD before transplantation, which arose predominantly from a country-specific effect in Germany. If patients with LVAD and specific risks such as infection receive Thymoglobulin induction plus early mTOR inhibition, the intensity of immunosuppression accumulates to an intolerable level with an associated increase in infection-related mortality. Such patients should not receive everolimus before wound healing is completed and any bacterial or fungal infection has been cleared.

The most important benefit of everolimus therapy in heart transplantation may be that its dual mode of action—prevention of acute allograft rejection coupled with suppression of growth factor-driven smooth muscle cell proliferation—combines immunosuppressive potency with the reduction of *de novo* CAV disease. The significant reduction in CMV infection in everolimus-treated patients may also contribute to the minimization of intimal vascular changes. For the first time, the A2310 study has shown superiority for everolimus versus MMF in all relevant IVUS parameters [9], in accordance with earlier subanalyses from the B253 study comparing everolimus with azathioprine [2]. Of note, everolimus was initiated early (i.e., within the first 72 hours after transplantation) in both trials. This may be an important detail as many preconditioning events which predispose to CAV start at the time of heart transplantation.

Careful patient selection and individualized immunosuppression are a key to achieving optimal outcomes after heart transplantation. Due to its potential to inhibit progression of CAV and to reduce CMV infection, everolimus should be initiated as soon as possible after heart transplantation and be included in standard immunosuppressive regimens if special care is applied in specific patient types and unsuitable patients are excluded (Table 5). Immediate and adequate reduction of CsA exposure is mandatory from the start of everolimus therapy. The findings of the MANDELA and SCHEDULE trials may, in the future, support adoption of CNI-free immunosuppression with combined everolimus and MMF therapy beyond six months after heart transplantation, and results of these trials are awaited with interest.

## Figures and Tables

**Table 1:** Clinical studies of everolimus versus azathioprine or mycophenolate mofetil (MMF) in *de novo* heart transplant recipients.

Study	Design	Primary endpoint	Everolimus	Comparator	CsA *C*_0_ target range (ng/mL) by month	Induction therapy	Steroids
B253 [2]	24-month Multicenter Randomized Double-blind for months 0–12, open-label for months 12–24	Composite efficacy failure at 6 months	Fixed-dose^a^ 1.5 mg/day (*n* = 209) 3.0 mg/day (*n* = 211)	Azathioprine^a^ 1–3 mg/kg/day (*n* = 214)	All 3 groups: 1: 250–400 2–6: 200–350 7–24: 100–300	In individual centers only: ATG or muromonab-CD3	Prednisolone, initiated at 0.5–1.0 mg/kg/day, tapered to achieve 0.3–0.5 mg/kg/day by day 21 and ≥0.1 mg/kg/day by month 6

Lehmkuhl et al. 2007 [10]	12-month Single center Retrospective	Not applicable	Initial dose before transplant 0.75 mg, then mean 1.5–1.75 mg/day, and *C*_0_ 3–8 ng/mL (*n* = 38)	MMF before transplant 1.0 g, then mean 1.5–2.5 g/day (*n* = 18)	Everolimus versus MMF: 1: 200–250, both groups 2: 175–200 versus 200–250 3-4: 150–175 versus 200–250 5-6: 100–150 versus 150–200 7–12: 80–120 versus 120–150	ATG 2.5 mg/kg/day on days 1 and 2	Initially, high-dose methylprednisolone, then prednisolone 1 mg/kg/day, tapered to achieve 0.1 mg/kg/day by month 12

A2411 [3]	12-month Multicenter Randomized Open-label	Noninferiority of renal function (calculated creatinine clearance at 6 months)	Initial dose^a^ 1.5 mg/day, *C*_0_ 3–8 ng/mL (*n* = 92)	MMF^a^ 3.0 g/day (*n* = 84)	Everolimus versus MMF: 1: 200–350, both groups 2: 150–250 versus 200–350 3-4: 100–200 versus 200–300 5-6: 75–150 versus 150–250 7–12: 50–100 versus 100–250	Antithymocyte antibodies (68.4% of patients) or IL-2RA (25.9% of patients)	Prednisone, tapered to achieve ≥0.1 mg/kg/day by month 6 and 0.1–0.05 mg/kg/day from month 6 to 12

A2310 [9]	24 months Multicenter Randomized Open-label	Noninferiority of composite efficacy failure at 12 months IVUS substudy: change in mean MIT at 12 months	Initial dose^a^ 1.5 mg/day, *C*_0_ 3–8 ng/mL (*n* = 282) or 3.0 mg/day, *C*_0_ 6–12 ng/mL (*n* = 168)	MMF^a^ 3.0 g/day (*n* = 271)	Everolimus versus MMF: 1: 200–350, both groups 2: 150–250 versus 200–350 3-4: 100–200 versus 200–300 5-6: 75–150 versus 150–250 7–24: 50–100 versus 100–250	Center-specific: No induction or Thymoglobulin or basiliximab	Yes, according to local practice

ATG: antithymocyte globulin; CsA: cyclosporine; IL-2RA: interleukin-2 receptor antibody; IVUS: intravascular ultrasound; MIT: maximum intimal thickness.

^a^First dose administered within 72 hours after transplant surgery.

**Table 2:** Efficacy endpoints in randomized trials of everolimus with reduced-exposure cyclosporine versus MMF with standard-exposure cyclosporine.

Parameter	A2310 [9]				A2411 [5]	
	12 months		24 months		12 months	
	MMF	Everolimus 1.5 mg	MMF	Everolimus 1.5 mg	MMF	Everolimus 1.5 mg
Number of patients	271	282	271	282	84	92
Composite efficacy failure^a^, %	33.6	35.1^b^	41.3	39.4^c^	41.7	32.6
AR associated with HDC, %	2.6	3.9	5.2	4.3	1.2	2.2
BPAR, ISHLT grade ≥ 3A, %	24.7	22.3	27.3	24.1	29.8	22.8^d^
BPAR treated with antibody, %	No data	No data	No data	No data	2.4	5.4
Graft loss/re-transplant, %	1.8	1.4	3.7	2.5	Composite: 11.9	Composite: 10.9
Death, %	4.8	7.8^e^	9.2	10.6^e^		
Loss to followup, %	3.7	3.2	5.2	3.5	No data	No data

AR: acute rejection; BPAR: biopsy proven acute rejection; HDC: hemodynamic compromise; ISHLT: International Society of Heart and Lung Transplantation; MMF: mycophenolate mofetil.

^a^Defined as BPAR grade ≥ 3A (or any BPAR in A2310), acute rejection associated with hemodynamic compromise, graft loss/retransplant, death, or loss to followup.

^b^ *P* = 0.002 for noninferiority (noninferiority margin 13%); *P* = 0.705 for no-difference test.

^c^Noninferior to the MMF group (noninferiority margin 13%).

^d^ *P* = 0.005 for noninferiority.

^e^Including one death in a patient who never received everolimus.

**Table 3:** Results of intravascular ultrasound (IVUS) substudies in randomized trials of everolimus with reduced-exposure cyclosporine versus MMF with standard-exposure cyclosporine.

Parameter	A2310 [9]			B253 [2, 20]		
	MMF	Everolimus 1.5 mg	*P* value	Azathioprine	Everolimus 1.5 mg/3.0 mg	*P* value
Number of patients						
12 months	101	88		72	70/69	
24 months	—	—		60	45/44	
Mean change in MIT from baseline, mm						
12 months	0.07 ± 0.11	0.03 ± 0.05	<0.00	0.10	0.04/0.03	0.01/0.003
24 months	—	—	1	0.15	0.07/0.06	0.014/0.004
Patients with CAV, %						
12 months	26.7	12.5	0.018	52.8	35.7/30.4	0.045/0.01
24 months	—	—		58.3	33.3/45.5	0.017/n.s.

CAV: cardiac allograft vasculopathy, defined as a change in MIT ≥ 0.5 mm as assessed by intravascular ultrasound (IVUS); MIT: maximal intimal thickness; MMF: mycophenolate mofetil.

**Table 4:** Patient selection for everolimus-based immunosuppression in *de novo* heart transplant recipients*.

Category	Remarks
Everolimus	
All *de novo* heart transplant recipients except for those with special conditions and/or risks (see the following)	Checking all patients for possibility of everolimus therapy due to its potential to reduce CNI-related toxicity, CMV infection, and malignancy risk and CAV

Everolimus only with special care	
Specific risks for renal impairment or creatinine increase	Reduceing CsA exposure to a minimum, monitoring urine electrophoresis and proteinuria, and stopping everolimus in the event of proteinuria > 1 g/day and/or signs of new glomerular damage on urine electrophoresis

Risks for wound healing disorders (diabetes mellitus, obese patients, high steroid exposure, and ventricular assist device)	Delay initiation of everolimus until completion of wound healing and resolution of any bacterial or fungal infection

Uncontrolled severe hyperlipidemia	Delay initiation of everolimus until serum lipids have been controlled Always administer everolimus in combination with lipid-lowering therapy for example, fluvastatin

Everolimus not appropriate	
(i) Paracorporal biventricular assist device with immanent risk of infection (ii) Infected ventricular assist device (iii) LVAD in conjunction with specific risks such as combination of Thymoglobulin induction and infection	Avoid antilymphocyte antibodies for induction in patients with elevated risk for early postoperative infection Everolimus may be initiated after completion of wound healing and resolution of any bacterial or fungal infection

Latent bacterial or fungal infections	Everolimus may be unsuitable in individual cases based on benefit/risk assessment

High probability of reoperation or necessity for additional surgery in the initial phase	Considering late initiation of everolimus to avoid the need to switch immunosuppressive regimen during a critical period

GFR < 40 mL/min/1.73 m^2^ if slope shows an ongoing deterioration of renal function	Delay initiation of everolimus Everolimus may be initiated if CNI exposure requires marked reduction

*Initiation within 72 hours after transplantation.

CAV: cardiac allograft vasculopathy; CNI: calcineurin inhibitor; CsA: cyclosporine; GFR: glomerular filtration rate; LVAD: left ventricular assist device.

**Table 5:** Overview of selected everolimus-associated adverse events.

Adverse event	Comment	Prevention/intervention
Dyslipidemia		Comedication with lipid-lowering medication is mandatory (statin not interacting with CYP450 e.g. fluvastatin, or fibrates)

Pancytopenia		In unexplained cytopenia (white blood cells, red cells, platelets), everolimus may be the cause and dose reduction or temporary cessation may be indicated

Acne		Improves within a few weeks using local treatment

Aphthous stomatitis		Local treatment is effective

Angioneurotic edema		Discontinue ACE inhibitor comedication

Creatine kinase (CK) elevation Muscle cramps	May be related to everolimus overexposure or/and to comedication of statin therapy	Everolimus trough concentration should be adjusted to the lower margin of the target range for several days and/or statin therapy should be stopped temporarily. If this is not effective, consider a temporary switch from everolimus to MMF In most cases, careful reintroduction of everolimus can be undertaken successfully after normalization of CK level and resolution of muscle cramps. Selected patients with persistent CK levels > 10-fold higher than normal may be referred for muscle biopsy [5]

Increased proteinuria	May reflect physiological tubular proteinuria due to mTOR inhibition, which is reversible and without clinical relevance as it does not reflect damage to renal tissue Proteinuria > 1 g/day indicates a glomerular process and may be due to an everolimus-associated event	Concomitant prescription of ACE inhibitor or angiotensin-receptor blockers may reduce the incidence of new onset proteinuria As proteinuria < 1 g/day does not exclude glomerular damage, urine protein electrophoresis can be performed to detect glomerular proteins

Noninfectious pneumonia	More likely to occur during sirolimus treatment in cancer patients	Requiring dose reduction or discontinuation and anti-inflammatory treatment by high-dose steroids. Frequent radiologic assessment is mandatory and laboratory values should be monitored twice a week

Impaired wound healing	Elevated risk early postoperatively in high-risk patients (e.g., diabetes, LVAD, redo surgery, and high-dose steroids) due to antiproliferative properties of mTOR inhibitors	Delayed onset of everolimus after transplant surgery, or temporary interruption during subsequent major surgery, may be helpful. In the event of minor local surgery in low-risk patients, everolimus therapy can be continued

Pericardial/pleural effusion	Elevated incidence early after heart transplantation	Manageable by frequent monitoring with echocardiography/sonography, symptomatic diuretic treatment, and drainage on demand

ACE: angiotensin converting enzyme; LVAD: left ventricular assist device; MMF: mycophenolate mofetil; mTOR: mammalian target-of-rapamycin inhibitor.
